# E2 level > 2950 pg/ml on hCG trigger day is an independent predictor for birthweight loss of full-term singletons born after fresh embryo transfers in non-PCOS patients

**DOI:** 10.1186/s12958-022-01027-9

**Published:** 2022-11-21

**Authors:** Jing Wu, Hengde Zhang, Xiaohong Wang

**Affiliations:** Reproductive Medicine Center, Department of Obstetrics and Gynecology, Tang Du Hospital, The Air Force Military medical University, 1 Xinsi Rd, Xi’an, 710038 Baqiao District China

**Keywords:** Oestradiol, hCG trigger day, Controlled ovarian stimulation, Full-term singleton, Small for gestational age (SGA)

## Abstract

**Background:**

Previous studies have demonstrated that the supraphysiological E2 level is negatively correlated with birthweight. However, the cut-off value of E2 level that significantly affects birthweight is unknown, and there is no definite conclusion regarding this level. Our study aimed to explore the threshold of the effect of E2 levels on birthweight.

**Design:**

A retrospective cohort study of 1846 samples was performed. All patients ≤42-years-old underwent autologous IVF cycles between August 1st, 2016 and April 30th, 2020. We categorized our data into four groups according to the E2 level: Group 1: ≤2000 pg/mL; Group 2: 2001–3000 pg/mL; Group 3: 3001–4000 pg/mL; and Group 4: > 4000 pg/mL.

**Results:**

The results of the multivariate regression analyses showed that when the E2 level was 3001–4000 pg/mL (adjusted β: − 89.64, 95% [CI]: − 180.29 to − 6.01; *P* = 0.0336) and greater than 4000 pg/mL (adjusted β: − 138.10, 95% [CI]: − 272.87 to − 10.33; *P* = 0.0181), weight loss was significant. Furthermore, the odds of full-term SGA were 1.40 times higher with E2 levels of 3001–4000 pg/mL (adjusted OR: 1.40, 95% [CI]: 1.090 to 3.18; *P* = 0.0256) and 2.55 times higher with E2 > 4000 pg/mL (adjusted OR: 2.55, 95% [CI]: 1.84 to 3.86; *P* = 0.0063) compared to the reference group. It can also be seen from the adjusted curves and the threshold effects that when the E2 level > 2950 pg/mL and > 3121 pg/mL, the incidence of SGA increased and the birthweight decreased, respectively.

**Conclusions:**

Our data suggest that E2 levels > 2950 pg/mL is an independent predictor for greater odds of full-term SGA singletons born after fresh embryo transfer.

**Supplementary Information:**

The online version contains supplementary material available at 10.1186/s12958-022-01027-9.

Assisted reproductive technology (ART) has rapidly advanced from the time that the first baby was born in 1978. An important advancement in in vitro fertilization (IVF) was the introduction of gonadotropin stimulation cycles to obtain more mature oocytes and more available embryos per cycle, thus increasing the cumulative live birth rate over a period of years [1]. However, it is well known that singleton pregnancies after fresh IVF cycles have a higher risk of obstetric and perinatal complications, such as low birth weight (LBW), small for gestational age (SGA), placenta previa, and preeclampsia, compared to those babies who are spontaneously born [2–7]. Some studies have suggested that the risks are the result of intrinsic factors in subfertile couples or ART itself, including controlled ovarian stimulation (COS), in vitro embryo culture, and cryopreservation techniques [1, 8–10]*.* However, it is difficult to know which step mainly contributes to the adverse effects.

During the COS process, supraphysiological levels of oestradiol (E2) can often be produced. In animal models, high levels of E2 have been reported to prematurely close the window of implantation and impede extravillous trophoblast invasion of the uterine spiral arteries, thus resulting in abnormal placentation-related complications, such as LBW or intrauterine growth restriction [11–13]. It is unclear whether this phenomenon exists in humans when pregnancies are achieved in a high E2 environment via COS in IVF fresh cycles. Previous studies have demonstrated that the hyperoestrogenic milieu produces a suboptimal uterine environment, which ultimately results in perinatal and neonatal complications [14–16]. However, the underlying mechanisms, whether due to the asynchronous development of the endometrium or abnormal extravillous trophoblast invasion, are still unknown. Imudia et al. reported that when an E2 level was more than 3450 pg/mL during fresh IVF-ET, the rates of SGA infants and preeclampsia were significantly increased [14]. Pereira et al. also reported 2.3 times higher odds of term LBW when the E2 level was > 3069.2 pg/mL in 2939 live singleton births of fresh IVF cycles [15]. In a later study by Pereira, it was suggested that E2 > 2500 pg/mL is an independent predictor for LBW in full-term singletons born to normal responder patients undergoing fresh cycles [16]. Previous studies have demonstrated that high E2 levels are harmful to neonatal health. However, it is unknown as to how high the cut-off value is for the E2 level. Obviously, there is currently no final conclusion. Therefore, our retrospective study aimed to explore the cut-off value of the E2 level on the hCG trigger day during COS in a model with full-term singletons born after autologous IVF fresh cycles in non-PCOS patients.

## Materials and methods

### Study design and population

We conducted a retrospective cohort study performed between August 1st, 2016 and April 30th, 2020 at the Center of Assisted Reproduction at Tangdu Hospital of Air Force Military Medical University in China. Our study population included all of the women who met the following criteria: autologous IVF/ICSI cycles, long luteal gonadotropin releasing hormone agonist (GnRH-a) protocol or antagonist protocol during the COS process, age ≤ 42 years at the time of oocyte retrieval, and having a full-term live singleton birth after fET. For the purpose of this study, patients with known polycystic ovarian syndrome (PCOS) who were diagnosed via the Rotterdam criteria were excluded from the analysis. The decision was made to prevent any basis of adverse birthweight outcomes associated with abnormal glucose and lipid metabolism, which is more likely to occur in PCOS. Furthermore, patients with multiple births, vanishing twins, uterine malformations, cervical incompetence, and a history of intrauterine and cervical surgery were also excluded.

A total of 1846 samples were included in this study. According to similar numbers of live births in each group, we categorized our data into four groups by dividing the total E2 level on the day of the hCG trigger: Group 1: ≤2000 pg/mL; Group 2: 2001–3000 pg/mL; Group 3: 3001–4000 pg/mL; and Group 4: > 4000 pg/mL.

### Ovarian stimulation and embryo transfer

The protocols of ovarian stimulation were achieved by using the GnRH-a long protocol or antagonist protocol. The starting dose and the type of COS protocol were determined by using patient characteristics and clinician preferences. All of the patients received both recombinant and urinary exogenous Gn. The daily Gn dose was decided on follicular growth in successive transvaginal sonograms and a blood test that included the evaluation of the plasma levels of E2, progesterone, and LH until the day of the hCG trigger. Ovulation was triggered in all of the patients when there were at least three follicles with ≥17 mm diameter on transvaginal ultrasound, with 250 μg of recombinant hCG (rhCG). Ultrasound-guided oocyte retrieval was performed 36 h after trigger injection. Approximately 12–17 h after insemination or sperm injection, the oocytes were examined for fertilization. Cleavage-stage embryos were transferred on the 3rd day, and blastocysts were transferred on the 5th day after oocyte retrieval [15]. There were no major changes in the clinical and laboratory conditions, culture media, or fET techniques during the study period.

### Study variables

Data on demographic and cycle characteristics were collected, including the ages of the couples, maternal body mass index (BMI), anti-Mullerian hormone (AMH) levels, basal follicle stimulating hormone (FSH) levels, type of infertility, prior Gn cycles, infertility duration, infertility cause, total Gn dose, length of stimulation, E2 levels on trigger day, the number of oocytes retrieved, the stages and numbers of transferred embryos, and the thickness and type of endometrium.

A full-term singleton was defined as a live birth at or after 37 weeks of gestation. The newborn height, weight, sex, gestational age (GA), and mode of delivery were recorded for all of the live infants. GA was counted from the day of embryo transfer, which was identified as Day 17 of the cycle for cleavage-stage embryo transfer and Day 19 for the blastocyst transfer [17]. LBW, very LBW, and foetal macrosomia were identified as birthweight < 2500 g, < 1500 g, and ≥ 4000 g, respectively. SGA and very SGA were identified as birthweight <10th and < 3rd percentiles. Large-for-gestational age (LGA) and very LGA were identified as birthweights >90th and > 97th percentiles. Birth weight percentiles were based on Chinese reference singleton newborns stratified by GA and neonatal sex [18].

### Statistical analysis

Categorical variables are expressed as the number of cases (n) with the percentage of occurrence (%), and continuous variables are expressed as the median (interquartile range [IQR]) or mean ± SD, as appropriate. The patient demographic, cycle characteristic, and neonatal outcomes were compared between the four groups via either *t* tests (for the continuous variables) or *X*^2^ tests (for the categorical variables).

A multiple linear regression analysis was performed to survey the relationship between the E2 level on the hCG trigger day and birthweight (g). A logistic regression analysis was introduced to assess the adverse categorical outcomes, such as LBW, very LBW, foetal macrosomia, SGA, very SGA, LGA, and very LGA, with adjustments for potential confounding factors. We selected the confounders on the basis of their associations with the outcomes of interest or a change in the effect estimate of more than 10%, including the ages of the couples, maternal BMI, basal FSH levels, AMH levels, prior gonadotropin cycle, COS protocols, total Gn doses, stimulation duration, fertilization method, number of retrieved oocytes, endometrial type, number and stage of the transferred embryos, and genders of the newborns.

All of the statistical analyses were performed by using EmpowerStats (www.empowerstats.com, X&Y solutions, Inc. Boston MA) and R software version 3.6.1 (http://www.r-project.org). A *P* value of < 0.05 was considered to be statistically significant.

## Results

A total of 1846 fresh IVF cycles that met the inclusion criteria during the study period were included in this analysis. Of these, 462, 486, 453, and 445 live-born singletons were categorized by Group 1 (≤2000 pg/mL), Group 2 (2001–3000 pg/mL), Group 3 (3001–4000 pg/mL), and Group 4 (> 4000 pg/mL), respectively.

The patient baseline characteristics, cycle parameters, and neonatal outcomes are presented in Table 1. Compared to Group 1, women in the higher E2 level groups were younger and had a lower mean BMI, lower basal FSH level, higher AMH level, and fewer prior gonadotropin cycles of the baseline characteristics. Furthermore, according to the cycle parameters, the higher E2 level groups had more GnRH-a long protocols, lower Gn doses, a longer stimulation length, more total retrieved oocytes, lower fertilization rates, and fewer numbers of cleavage-stage embryos that were transferred. In terms of neonatal outcomes, with increasing E2 levels on the hCG trigger day, the weight of full-term newborns gradually decreased, the proportion of low birth-weight infants and the incidence of SGA babies increased, and the rate of caesarean sections also decreased. The differences in all of the above mentioned indices were significant (*P* < 0.001).

**Table 1:** Patient clinical characteristics, cycle parameters and neonatal outcomes by different E2 level on hCG trigger day

Characteristics	≤2000 ng/ml	2001–3000 ng/ml	3001–4000 ng/ml	>4000 ng/ml	***P*** value
	(***n*** = 462)	(***n*** = 486)	(***n*** = 453)	(***n*** = 445)	
**Baseline characteristics**					
**Maternal age (y)**	32.05 ± 4.12	30.94 ± 3.90	30.45 ± 3.84	30.18 ± 3.67	< 0.001
**Paternal age (y)**	33.64 ± 5.01	32.53 ± 4.85	31.88 ± 4.52	31.78 ± 4.46	< 0.001
**Maternal BMI (kg/m**^**2**^**)**	22.64 ± 3.01	22.53 ± 3.34	21.97 ± 2.85	21.83 ± 3.18	< 0.001
**Basal FSH (IU/L)**	8.98 ± 3.19	7.53 ± 2.32	7.10 ± 1.75	6.99 ± 1.73	< 0.001
**AMH (ng/ml)**	1.62 ± 1.79	2.63 ± 2.00	3.52 ± 2.34	3.76 ± 2.17	< 0.001
**Type of infertility**					0.145
Primary	216 (46.75%)	261 (53.70%)	224 (49.45%)	233 (52.36%)	
Secondary	246 (53.25%)	225 (46.30%)	229 (50.55%)	212 (47.64%)	
**Infertility duration (y)**	3.89 ± 2.85	3.58 ± 2.63	3.54 ± 2.60	3.59 ± 2.68	0.166
**Infertility cause**					0.377
Female	314 (67.97%)	304 (62.55%)	290 (64.02%)	288 (64.72%)	
Male	80 (17.32%)	105 (21.60%)	88 (19.43%)	89 (20.00%)	
Mixed	45 (9.74%)	44 (9.05%)	39 (8.61%)	47 (10.56%)	
Unexplained	23 (4.98%)	33 (6.79%)	36 (7.95%)	21 (4.72%)	
**Prior gonadotropin cycle**	1.42 ± 0.76	1.19 ± 0.45	1.13 ± 0.40	1.13 ± 0.44	< 0.001
**Ovarian stimulation parameters**					
**COS protocols**					< 0.001
GnRH-a long protocol	196 (42.42%)	300 (61.73%)	345 (76.16%)	370 (83.15%)	
Antagnist protocol	266 (57.58%)	186 (38.27%)	108 (23.84%)	75 (16.85%)	
**Dosage of gonadotropins (IU)**	2748.01 ± 1110.08	2407.71 ± 1118.06	2118.14 ± 1113.68	1807.56 ± 870.33	< 0.001
**Stimulation duration (days)**	10.82 ± 2.33	11.53 ± 2.03	12.05 ± 2.18	11.90 ± 2.06	< 0.001
**E2 level on HCG day (pg/ml)**	1413.19 ± 439.51	2493.55 ± 293.11	3477.02 ± 290.95	4697.74 ± 521.27	< 0.001
**Number of oocytes retrieved**	6.09 ± 3.00	9.48 ± 2.94	11.11 ± 3.06	12.18 ± 3.21	< 0.001
**Retrieved MII Oocytes**	5.30 ± 2.59	8.04 ± 2.64	9.49 ± 2.95	10.57 ± 3.20	< 0.001
**Fertilization method**					0.180
IVF	319 (69.05%)	308 (63.37%)	294 (64.90%)	297 (66.74%)	
ICSI	128 (27.71%)	145 (29.84%)	132 (29.14%)	130 (29.21%)	
IVF + ICSI	15 (3.25%)	33 (6.79%)	27 (5.96%)	18 (4.04%)	
**Fertilization rate**	85.10 ± 17.35	83.29 ± 15.39	81.58 ± 16.48	82.39 ± 15.86	0.008
**Number of available embryos**	3.07 ± 1.63	4.37 ± 2.20	4.96 ± 2.24	5.72 ± 2.51	< 0.001
**Stage embryo transferred**					< 0.001
D3	429 (92.86%)	412 (84.77%)	363 (80.13%)	338 (75.96%)	
D5	33 (7.14%)	74 (15.23%)	90 (19.87%)	107 (24.04%)	
**Number of embryos transferred**					0.011
1	120 (25.97%)	108 (22.22%)	131 (28.92%)	139 (31.24%)	
2	339 (73.38%)	377 (77.57%)	322 (71.08%)	306 (68.76%)	
3	3 (0.65%)	1 (0.21%)	0 (0.00%)	0 (0.00%)	
**Endometrial thickness (mm)**	9.81 ± 1.51	10.07 ± 1.57	9.97 ± 1.56	10.03 ± 1.48	0.057
**Endometrial type**					0.388
A	5 (1.08%)	11 (2.26%)	5 (1.10%)	7 (1.57%)	
A-B	24 (5.19%)	22 (4.53%)	29 (6.40%)	29 (6.52%)	
B	149 (32.25%)	151 (31.07%)	128 (28.26%)	136 (30.56%)	
B-C	252 (54.55%)	269 (55.35%)	268 (59.16%)	234 (52.58%)	
C	32 (6.93%)	33 (6.79%)	23 (5.08%)	39 (8.76%)	
**Neonatal outcomes indicators**					
**Gender of newborn**					1.000
Male	232 (50.22%)	244 (50.21%)	228 (50.33%)	223 (50.11%)	
Female	230 (49.78%)	242 (49.79%)	225 (49.67%)	222 (49.89%)	
**Newborn height**	50.37 ± 1.73	50.40 ± 1.67	50.37 ± 1.88	50.54 ± 1.67	0.388
**GA (week)**	39.32 ± 0.99	39.38 ± 1.12	39.38 ± 1.14	39.38 ± 1.00	0.744
**Birthweight (g)**	3440.94 ± 449.91	3438.92 ± 388.16	3366.19 ± 447.61	3322.45 ± 415.85	0.021
**Z-score**	0.14 ± 1.14	0.13 ± 0.95	− 0.05 ± 1.10	−0.12 ± 0.97	0.004
**Birthweight**					0.040
Normal birthweight	418 (90.48%)	450 (92.59%)	406 (89.62%)	400 (89.89%)	
Very low birth weight(<1500 g)	0 (0.00%)	0 (0.00%)	0 (0.00%)	5 (1.12%)	
Low birthweight(<2500 g)	9 (1.95%)	1 (0.21%)	10 (2.21%)	15 (3.37%)	
Fetal macrosomia(≥4000 g)	35 (7.58%)	35 (7.20%)	37 (8.17%)	25 (5.62%)	
**Small for gestational age**	31 (6.71%)	26 (5.35%)	38 (8.39%)	42 (9.44%)	0.020
**Very small for gestational age**	12 (2.60%)	3 (0.62%)	9 (1.99%)	10 (2.25%)	0.114
**Large for gestational age**	41 (8.87%)	44 (9.05%)	46 (10.15%)	29 (6.52%)	0.263
**Very large for gestational age**	16 (3.46%)	10 (2.06%)	20 (4.42%)	10 (2.25%)	0.125
**Mode of delivery**					0.028
Vaginal	140 (30.30%)	182 (37.45%)	161 (35.54%)	175 (39.33%)	
Caesarean section	322 (69.70%)	304 (62.55%)	292 (64.46%)	270 (60.67%)	

The univariate linear analysis shown in Table 2 revealed that seven factors significantly influenced birthweight, including maternal BMI (unadjusted β: 15.97, 95% [CI]: 9.79 to 22.15; *P* < 0.0001), COS protocols (unadjusted β: − 52.70, 95% [CI]: − 94.43 to − 10.96; *P* = 0.0134), stimulation duration (unadjusted β: 9.36, 95% [CI]: 0.55 to 18.18; *P* = 0.0375), E2 levels on HCG day (100 pg/mL) (unadjusted β: − 5.49, 95% [CI]: − 8.83 to − 1.23; *P* = 0.023), fertilization method (unadjusted β: − 92.14, 95% [CI]: − 182.74 to − 1.54; *P* = 0.0464), endometrial type (unadjusted β: 183.22, 95% [CI]: 9.24 to 357.19; *P* = 0.0391), and newborn sex (unadjusted β: − 109.08, 95% [CI]: − 147.60 to − 70.56; *P* < .0001).

**Table 2:** Univariate liner analysis of impact factors on birthweight (*n* = 1846)

Exposure	Values mean ± SD / n (%)	Change in birthweight(g) β(95%CI)	***P*** value
**Maternal age (y)**	30.92 ± 3.95	−1.20 (−6.12, 3.73)	0.6338
**Paternal age (y)**	32.46 ± 4.77	−0.37 (−4.44, 3.70)	0.8571
**Maternal BMI (kg/m**^**2**^**)**	22.25 ± 3.12	15.97 (9.79, 22.15)	< 0.0001
**Basal FSH**	7.66 ± 2.46	−1.05 (− 8.95, 6.85)	0.7948
**AMH**	2.87 ± 2.24	5.09 (−3.57, 13.75)	0.2495
**Type of infertility**			
Primary	934 (50.60%)	Ref	
Secondary	912 (49.40%)	30.32 (−8.50, 69.14)	0.1260
**Infertility duration (y)**	3.65 ± 2.69	−6.65 (−13.85, 0.56)	0.0708
**Infertility cause**			
Female	1196 (64.79%)	Ref	
Male	362 (19.61%)	−27.00 (−77.04, 23.05)	0.2906
Mixed	175 (9.48%)	25.37 (−42.16, 92.89)	0.4616
Unexplained	113 (6.12%)	−34.34 (− 116.45, 47.77)	0.4125
**Prior gonadotropin cycle**	1.22 ± 0.54	−9.29 (−44.99, 26.41)	0.6101
**COS protocols**			
GnRH-a long protocol	1211 (65.60%)	Ref	
Antagnist protocol	635 (34.40%)	−52.70 (−94.43, −10.96)	0.0134
**Dosage of gonadotropins (IU)**	2277.14 ± 1114.47	0.02 (−0.00, 0.03)	0.0764
**Stimulation duration (days)**	11.57 ± 2.20	9.36 (0.55, 18.18)	0.0375
**E2 level on HCG day (100 pg/ml)**	29.96 ± 12.67	−5.49 (−8.83, −1.23)	0.023
**Number of oocytes retrieved**	9.68 ± 3.81	2.63 (− 2.47, 7.72)	0.3120
**Fertilization method**			
IVF	1218 (65.98%)	Ref	
ICSI	535 (28.98%)	−29.85 (−73.09, 13.39)	0.1762
IVF + ICSI	93 (5.04%)	−92.14 (−182.74, − 1.54)	0.0464
**Stage embryo transferred**			
D3	1542 (83.53%)	Ref	
D5	304 (16.47%)	13.04 (−39.32, 65.40)	0.6255
**Number of embryos transferred**			
1	498 (26.98%)	Ref	
2	1344 (72.81%)	22.23 (−21.54, 66.00)	0.3197
3	4 (0.22%)	141.86 (− 276.99, 560.71)	0.5069
**Endometrial thickness (mm)**	9.97 ± 1.53	8.59 (−4.09, 21.27)	0.1844
**Endometrial type**			
A	28 (1.52%)	Ref	
A-B	104 (5.63%)	54.59 (− 122.82, 232.01)	0.5465
B	564 (30.55%)	91.85 (−69.49, 253.20)	0.2646
B-C	1023 (55.42%)	106.98 (−52.65, 266.60)	0.1892
C	127 (6.88%)	183.22 (9.24, 357.19)	0.0391
**Gender of newborn**			
Male	927 (50.22%)	Ref	
Female	919 (49.78%)	−109.08 (− 147.60, − 70.56)	< 0.0001

Ref, reference group

The birthweight outcomes of the multivariate analyses are shown in Table 3. The birthweight appeared to be negatively associated with the increasing E2 concentration on the hCG trigger day (adjusted β: − 6.154, 95% [CI]: − 10.62 to − 2.29; *P* = 0.0018), even after accounting for the confounding variables. The effective value β implied that for every 100 pg/mL increase in the E2 concentration, birthweight decreased by 6.154 g. Moreover, when the E2 level was taken as the categorical indicator, the birthweight still exhibited a declining trend by the increase in the E2 level in the four groups (3438.61 g vs. 3426.49 g vs. 3348.97 g vs. 3300.51 g, P trend = 0.012). Especially when the E2 level was 3001–4000 pg/mL (adjusted β: − 89.64, 95% [CI]: − 180.29 to − 6.01; *P* = 0.0336) and greater than 4000 pg/mL (adjusted β: − 138.10, 95% [CI]: − 272.87 to − 10.33; *P* = 0.0181), weight loss was significant compared to the group of less than 2000 pg/mL. Furthermore, full-term SGA was significantly increased with increasing E2 levels (adjusted OR: 1.030, 95% [CI]: 1.010 to 1.052; *P* = 0.0038). When the E2 level was categorized into four groups, the odds of full-term SGA were 1.40 times higher with E2 levels of 3001–4000 pg/mL (adjusted OR: 1.40, 95% [CI]: 1.090 to 3.18; *P* = 0.0256) and 2.55 times higher with E2 > 4000 pg/mL (adjusted OR: 2.55, 95% [CI]: 1.84 to 3.86; *P* = 0.0063) compared to the reference group (E2 ≤ 2000 pg/mL). However, there was no significant difference in the rate of LBW, very LBW, foetal macrosomia, very SGA, LGA, or very LGA.

**Table 3:** Crude and adjusted odds ratios/β for the effect of E2 level (100 pg/ml) on birthweight outcomes

Characteristics	Birthweight (g) Adjust mean (95%CI)	Crude OR/β (95% CI)	***P*** value	*Adjust OR/β (95% CI)	***P*** value
**Birthweight (g)**					
**E2 level on hCG trigger day**		−5.49 (−8.83, −1.23)	0.023	**− 6.154 (− 10.62,-2.29)**	**0.0018**
**E2 level on hCG trigger day (catergorized into four groups)**					
≤2000 pg/ml(n = 462)	3438.61 (3379.60, 3507.61)	Ref		Ref	
2001–3000 pg/ml(n = 486)	3426.49 (3378.02, 3474.97)	−1.98 (−74.23, 71.19)	0.5393	−12.11 (− 92.94, 68.72)	0.2964
3001–4000 pg/ml(*n* = 453)	3348.97 (3293.26, 3404.68) *	−73.75 (− 160.91, 29.42)	0.8382	**−89.64 (− 180.29, − 6.01)**	**0.0336**
>4000 pg/ml(*n* = 445)	3300.51 (3256.33, 3344.68) *	−118.49 (− 240.90, 6.92)	0.3137	**−138.10 (− 272.87, − 10.33)**	**0.0181**
**P trend**	**0.012**				
**Other outcomes**					
**SGA**		0.998 (0.984, 1.012)	0.81620	**1.030 (1.010, 1.052)**	**0.0038**
**SGA (catergorized into four groups)**					
≤2000 pg/ml(n = 462)		Ref		Ref	
2001–3000 pg/ml(n = 486)		0.84 (0.52, 1.37)	0.4838	0.81 (0.46, 1.41)	0.4497
3001–4000 pg/ml(n = 453)		1.02 (0.54, 1.62)	0.6015	**1.40 (1.09, 3.18)**	**0.0256**
>4000 pg/ml(n = 445)		1.61 (0.87, 2.42)	0.0618	**2.55 (1.84, 3.86)**	**0.0063**
**LBW**		1.005 (0.975, 1.035)	0.75434	1.038 (0.995, 1.083)	0.08415
**Very LBW**		1.09 (0.98, 1.22)	0.1022	1.20 (0.86, 1.67)	0.2805
**Fetal macrosomia**		1.00 (0.98, 1.01)	0.5212	0.99 (0.97, 1.01)	0.3326
**Very SGA**		1.00 (0.97, 1.02)	0.7879	1.04 (1.00, 1.08)	0.0681
**LGA**		0.99 (0.98, 1.01)	0.3898	0.99 (0.97, 1.01)	0.2540
**Very LGA**		1.00 (0.98, 1.02)	0.9559	0.99 (0.96, 1.02)	0.4932

*Analyses were adjusted for couple’s age, maternal BMI, basal FSH, AMH, prior gonadotropin cycle, COS protocols, total Gn dose, stimulation duration, fertilization method, number of oocytes retrieved, endometrial type, number and stage of embryos transferred, newborn sex

Adjust OR = adjusted odds ratio; CI = confifidence interval

Ref, reference group

**Fig. 1:**
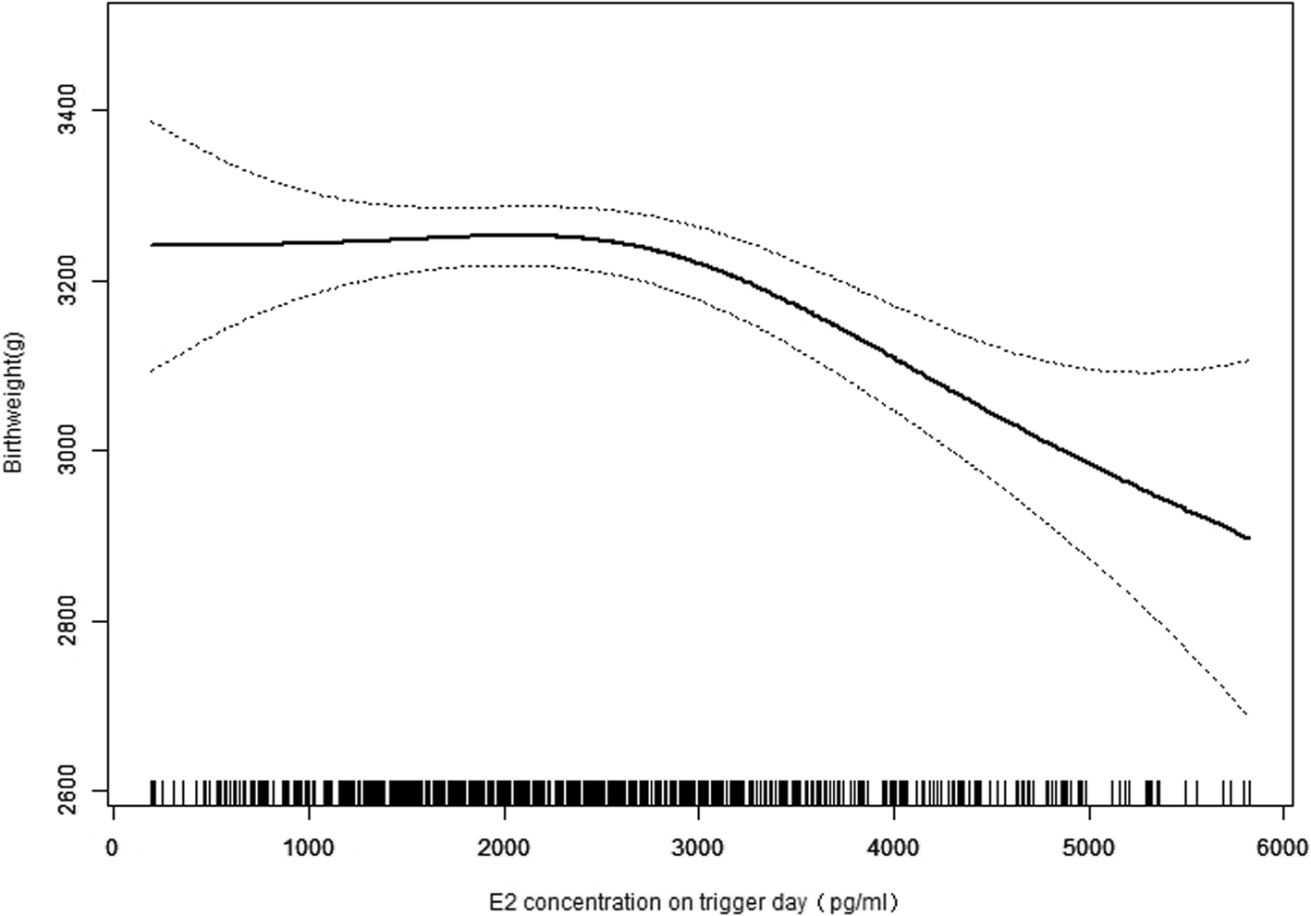
The relationship between E2 concentration (pg/ml) on hCG trigger day and adjust mean birthweight(g)

**Fig. 2:**
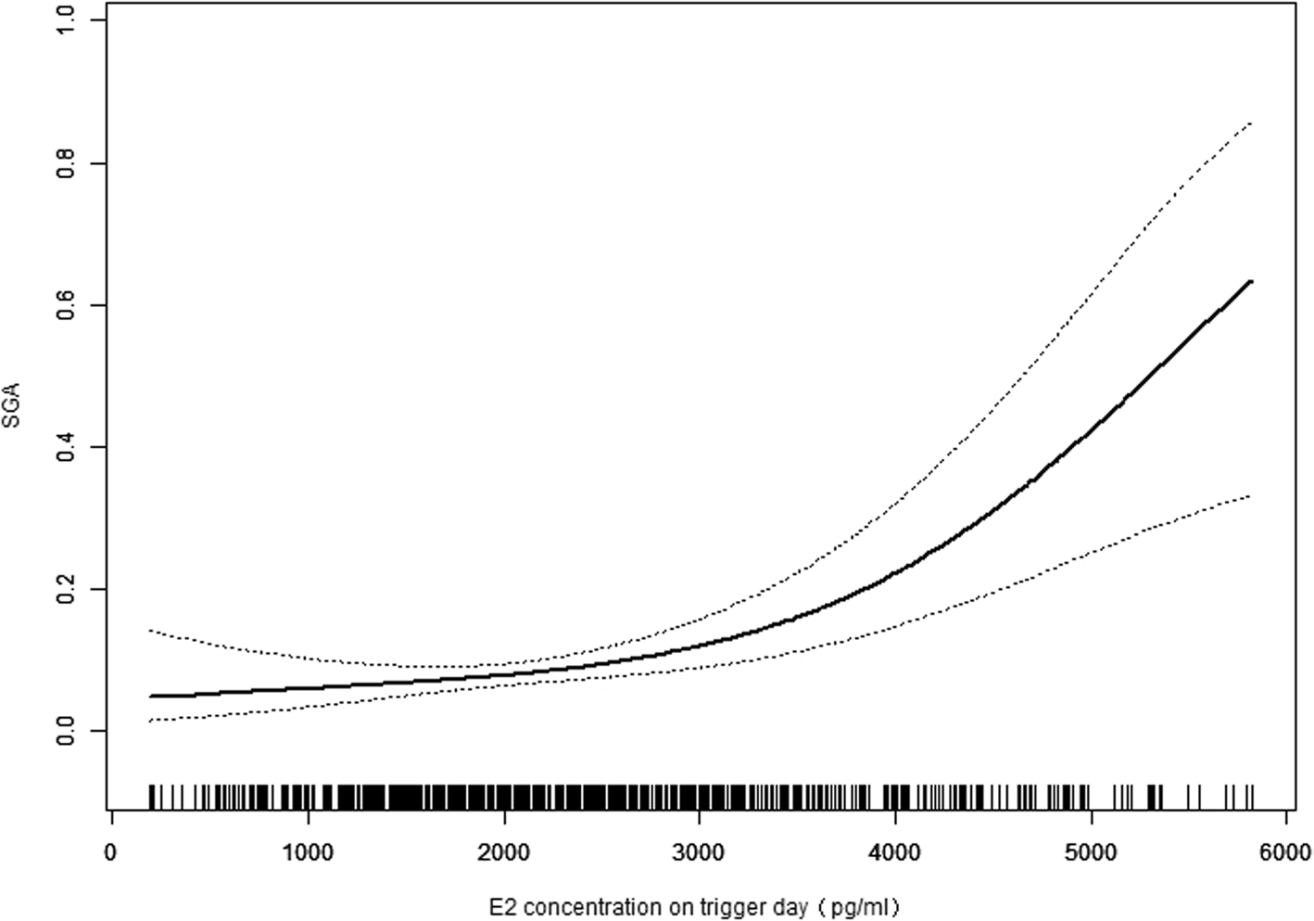
The relationship between E2 concentration (pg/ml) on hCG trigger day and adjust of SGA *Analyses of both in Fig. 1 and Fig. 2 were adjusted for couple’s age, maternal BMI, basal FSH, AMH, prior gonadotropin cycle, COS protocols, total Gn dose, stimulation duration, fertilization method, number of oocytes retrieved, endometrial type, number and stage of embryos transferred, newborn sex

The curves in Figs. 1 and 2 show the relationship between the E2 concentration on the hCG trigger day and the adjusted mean birthweight and SGA incidence, respectively. Combined with the threshold effect of the E2 level (100 pg/mL) on birthweight outcomes by using piecewise linear regression displayed in Table 4, it indicated that when the E2 level > 2950 pg/mL, SGA incidence increases were obvious (adjusted OR: 1.163, 95% [CI]: 1.073 to 1.325; *P* = 0.0027). When the E2 level was > 3121 pg/mL, birthweight loss (adjusted β: − 15.77, 95% [CI]: − 23.87 to − 7.67; *P* = 0.0001) was significant. This may indicate that 2950 pg/mL is the inflection point of the E2 level on the hCG trigger day affecting the outcome of birthweight.

**Table 4:** Threshold effect of E2 level (100 pg/ml) on birthweight outcomes using piece-wise linear regression

Characteristics	Crude β/OR (95% CI)	***P*** value	^a^Adjust β/OR (95% CI)	***P*** value
**SGA**				
≤29.50	0.927 (0.614, 1.477)	0.623	1.002 (0.819, 1.152)	0.281
> 29.50	1.031 (1.014, 1.075)	0.029	**1.163 (1.073, 1.325)**	**0.0027**
**Birthweight (g)**				
≤31.21	1.90 (−3.94, 7.74)	0.5236	−2.94 (−11.75, 5.86)	0.5123
> 31.21	−15.14 (−22.30, −7.98)	< 0.0001	**− 15.77 (− 23.87, − 7.67)**	**0.0001**

^a^Analyses were adjusted for couple’s age, maternal BMI, basal FSH, AMH, prior gonadotropin cycle, COS protocols, total Gn dose, stimulation duration, fertilization method, number of oocytes retrieved, endometrial type, number and stage of embryos transferred, newborn sex

## Discussion

This current retrospective cohort study analysed 1846 infertile women under 42-years-old with full-term live singleton births conceived via fresh IVF-ET. Our findings indicated that the E2 level on the hCG trigger day was negatively correlated with birthweight. The effective value indicated that for every 100 pg/mL increase in E2 concentration, birthweight decreased by 6.154 g. After grouping the E2 levels, the analysis also confirmed the negative relationship, especially when the E2 level was 3001–4000 pg/mL, the birthweight demonstrated a loss of 89.64 g; when the E2 level was more than 4000 pg/mL, the birthweight was reduced by 138.10 g compared to the group of less than 2000 pg/mL. It is also important to note that during in vitro fertilization, the superbiological hormone environment of COS during implantation is associated with the high probability of SGA infant birth. The odds of full-term SGA were 1.40 times higher with E2 levels of 3001–4000 pg/mL and 2.55 times higher with E2 > 4000 pg/mL compared to the reference group. The results of curve fitting and threshold effect showed the specific cut-off value. When E2 levels were greater than 2950 pg/mL, the incidence of SGA increased significantly, whereas when E2 levels were greater than 3121 pg/mL, the birthweight began to decrease. This may suggest that E2 levels > 2950 pg/mL is an independent risk factor for greater odds of full-term SGA singletons born after fET in non-PCOS patients.

Oestradiol has been shown to play a key role in the morphological and functional differentiation of trophoblasts, and the regulation of uteroplacental blood flow is essential for the optimal foetal growth and development of nonhuman primate pregnancies [19, 20]. In a mouse model, Ertzeid et al. observed in 2001 that the average birth weight of offspring after blastocyst transfer of superovulation was lower than that without stimulated embryo transfer [21]. It was also reported that the foetal weight of superovulated mice was 25% less than that of the control group [22]. In fact, hyperstimulation may affect foetal growth by altering the remodelling of spiral arteries and the invasion of trophoblasts in mice [23]. Some researchers have suggested that the reasons for this effect involve the increase in umbilical artery resistance and the decrease in placental microvessel density [24], and other researchers have considered that the impairment in placentation may be the result of E2-induced differential expression of the Grb10 gene [25] and the GATA3 transcription factor [26]. This mechanism has been fully elucidated in animal models. It is speculated that the aforementioned findings in mouse models could explain some of the clinical findings of LBW associated with fresh IVF-ET in human-based studies.

In humans, some studies have shown that there is no difference in the mean total birth weight or the incidence of LBW, SGA, or preterm birth (PTB) based on different E2 levels [27–29]. However, more observational evidence suggests that high E2 levels in fET are closely related to adverse perinatal and neonatal outcomes, as shown in our current study. This implied that COS can affect the endometrial environment during implantation, as well as impair the process of placental formation and thus impact the obstetric outcome via excessive physiological levels of E2. The specific mechanism may be similar to that of the abovementioned animal models. However, there is a question as to how much higher the E2 level must be to be harmful for newborns.

Different studies have obtained different conclusions on the cut-off value of the E2 level for the effect on birthweight. In a recent study by Pereira et al., 4071 patients < 40-years-old with live singleton births were included, and all of the patients who had PCOS were excluded, which was similar to the inclusion criteria in our analysis. The results showed that an increased risk of full-term LBW was demonstrated when E2 levels reached more than 2500 pg/mL on the trigger day compared to the group of E2 levels 500–1500 pg/mL, and this risk increased considerably when E2 levels exceeded 4000 pg/mL undergoing fresh cycles [16], thus suggesting that E2 levels > 2500 pg/mL is an independent predictor for LBW in full-term singletons after fET. A prior study by Pereira with 2939 live singleton births also reported 2.3 times higher odds of full-term LBW singletons when the E2 level was greater than 3069.2 pg/mL [15]. However, it is unclear why the results differ between the studies by Pereira and our study. An earlier study of 292 live singleton births conceived with fresh IVF-ET found that E2 levels > 3450 pg/mL on the trigger day were associated with a higher risk of developing preeclampsia and SGA infants [14], which was similar to our results. However, unlike our study, this article also analysed the data of preeclampsia, which revealed that the greater odds of SGA were closely related to preeclampsia during pregnancy. However, the selection criteria were not comprehensive. For example, only multiple live births were excluded, and there was no restriction on patient age or the ratio of PCOS women who were prone to abnormal glucose and lipid metabolism, which was more likely to lead to adverse neonatal outcomes.

In our study, to pursue research on birthweight outcomes, only patients younger than 42-years-old who obtained singleton births with a single gestational sac were included, and PCOS patients were excluded. The results showed that serum E2 levels on the hCG trigger day were negatively associated with birthweight and positively correlated with the incidence of SGA. In particular, when the E2 level was > 3121 pg/mL and > 2950 pg/mL, the loss of birthweight and the increased influence of SGA infants were significant. Based on the abovementioned results, it can be seen that the cut-off value of the effect for E2 level on birthweight is approximately 3000 pg/mL. However, there is a question as to why the cut-off value is approximately 3000 pg/mL. Royster et al. revealed a relationship between the increasing incidence of adverse placental complications (such as placenta accreta or placenta previa) when E2 levels were greater than 3000 pg/mL on the trigger day [30]. As mentioned above, oestradiol has been shown to be an important regulator in villous trophoblastic development and uteroplacental blood flow. However, excessive levels of E2 may affect trophoblast invasion and placental development, thus threatening maternal and foetal safety.

YF Ying et al. has shown that the E2 level on hCG day may be related to the incidence of ovarian hyperstimulation syndrome (OHSS) [31]. So, will high E2 level affect birthweight through the high incidence of OHSS? Most studies have reported that there was no correlation between the incidence of OHSS and the weight of newborns after IVF [32–34]. OHSS, which occurs in the luteal phase or early pregnancy in IVF patients and represents abnormal transient hemodynamics, does not exert any obviously adverse effect on the subsequent pregnancy [34]. Therefore, in our study the incidence of OHSS was not included in the confounding factors.

The main strength of the study included its demonstration that supraphysiological levels of E2 are an independent indicator for birthweight loss and for SGA of full-term singletons after fresh IVF-ET cycles in non-PCOS patients. The limitation of our current study was that we are aware of significant differences in baseline data and ovarian stimulation parameters between the four groups, including patient age, BMI, basal FSH, AMH, prior Gn cycles, the selection of dose and protocol based on patient characteristics and clinician preferences, length of the cycle and appearance of endometrium. In order to explore the independent effect of E2 level on birthweight, we made accurate statistical correction for these confounding factors as far as possible in each regression analysis. However, it is still not ruled out that the internal factors of patients cause bias to the outcome. A second limitation was that we did not adjust for pregnancy complications, such as hypertensive disorder, gestational diabetes mellitus, or placental dysfunctions, because these variables were not collected in the case record forms of the original trials, which may have resulted in bias.

Prospective randomized control trials with a larger sample size and continuous follow-up from pregnancy to birth are needed to further validate the exact effects of supraphysiological E2 levels in the COS process on birthweight outcomes.

In conclusion, in full-term singletons of non-PCOS patients after fET, serum E2 levels on the hCG trigger day are negatively associated with birthweight. In particular, when the E2 level was over 2950 pg/mL, the odds of full-term SGA singletons began to significantly increase. Our data suggest that E2 > 2950 pg/mL may be an independent risk factor for birthweight loss and for greater odds of full-term SGA singletons born after fET in non-PCOS patients. Therefore, in recent years, conservative, step-down, and mild ovarian stimulation protocols have been highlighted and advocated for this purpose.

## Supplementary Information

**Additional file 1.**

## Data Availability

The datasets analysed during the current study available from the corresponding author on reasonable request.

## Abbreviations

ART: Assisted reproductive technology
E2: Oestradiol
IVF-ET: In vitro fertilization-embyro trasfer
fET: Fresh embryo transfer
BMI: Maternal body mass index
AMH: Anti-Mullerian hormone
FSH: Follicle stimulating hormone
Gn: Gonadotropin
COS: Controlled ovarian stimulation
PCOS: Polycystic ovarian syndrome
HCG: Human chorionic gonadotropin
SGA: Small for gestational age
LBW: Low birth weight
