# Supplementary figures and images for: E2 level > 2950 pg/ml on hCG trigger day is an independent predictor for birthweight loss of full-term singletons born after fresh embryo transfers in non-PCOS patients
Source: Reprod Biol Endocrinol. 2022 Nov 21;20:162. doi: 10.1186/s12958-022-01027-9 (PMC9677889; doi:10.1186/s12958-022-01027-9)

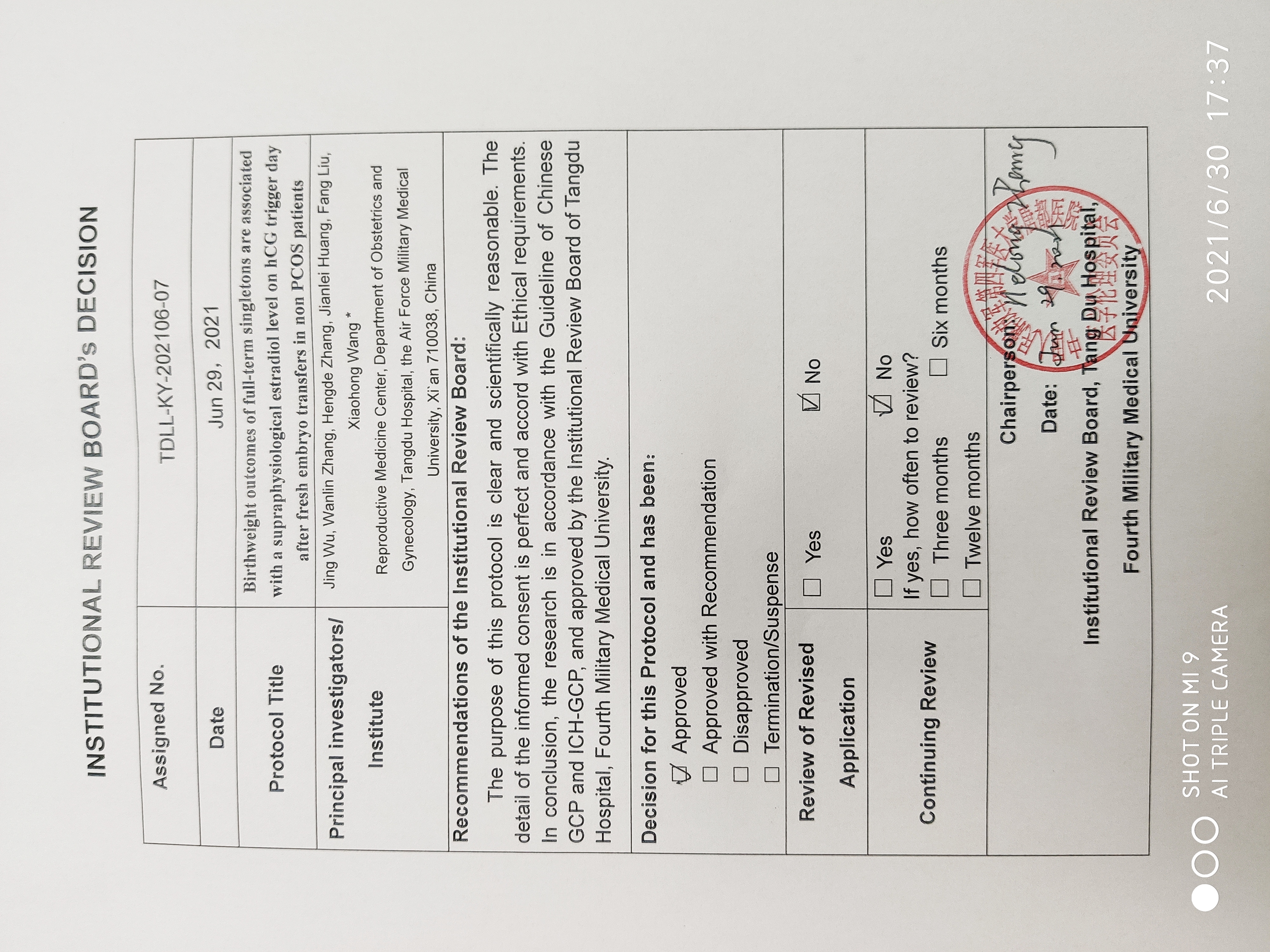

Supplement: Supplementary file 1 — Additional file 1. [file 12958_2022_1027_MOESM1_ESM.jpg]
